# Whole exome sequencing identifies deleterious rare variants in *CCDC141* in familial self-limited delayed puberty

**DOI:** 10.1038/s41525-021-00274-w

**Published:** 2021-12-20

**Authors:** Tansit Saengkaew, Gerard Ruiz-Babot, Alessia David, Alessandra Mancini, Katia Mariniello, Claudia P. Cabrera, Michael R. Barnes, Leo Dunkel, Leonardo Guasti, Sasha R. Howard

**Affiliations:** 1 Centre for Endocrinology, William Harvey Research Institute, Barts and the London School of Medicine and Dentistry, Queen Mary University of London, London, UK; 2 Endocrinology Unit, Department of Paediatrics, Faculty of Medicine, Prince of Songkla University, Songkhla, Thailand; 3 Department of Life Sciences, Centre for Integrative Systems Biology and Bioinformatics, Imperial College London, London, UK; 4 Centre for Translational Bioinformatics, William Harvey Research Institute, Barts and the London School of Medicine and Dentistry, Queen Mary University of London, London, UK; 5 NIHR Barts Cardiovascular Biomedical Research Centre, Barts and The London School of Medicine and Dentistry, Queen Mary University of London, London, UK

**Keywords:** Next-generation sequencing, Gene regulation, Molecular medicine, Translational research, Paediatric research

## Abstract

Developmental abnormalities of the gonadotropin-releasing hormone (GnRH) neuronal network result in a range of conditions from idiopathic hypogonadotropic hypogonadism to self-limited delayed puberty. We aimed to discover important underlying regulators of self-limited delayed puberty through interrogation of GnRH pathways. Whole exome sequencing (WES) data consisting of 193 individuals, from 100 families with self-limited delayed puberty, was analysed using a virtual panel of genes related to GnRH development and function (*n* = 12). Five rare predicted deleterious variants in *Coiled-Coil Domain Containing 141* (*CCDC141*) were identified in 21 individuals from 6 families (6% of the tested cohort). Homology modeling predicted all five variants to be deleterious. CCDC141 mutant proteins showed atypical subcellular localization associated with abnormal distribution of acetylated tubulin, and expression of mutants resulted in a significantly delayed cell migration, demonstrated in transfected HEK293 cells. These data identify mutations in *CCDC141* as a frequent finding in patients with self-limited delayed puberty. The mis-localization of acetylated tubulin and reduced cell migration seen with mutant CCDC141 suggests a role of the CCDC141-microtubule axis in GnRH neuronal migration, with heterozygous defects potentially impacting the timing of puberty.

## Introduction

Puberty is an intricate biological process, which results from the activation of the hypothalamic-pituitary-gonadal (HPG) axis. Before this axis is established, GnRH neurons undergo a complex migratory journey from the nasal placode into the hypothalamus during fetal life. An elaborate interaction of paracrine signaling networks influences both this migratory process and future GnRH secretion^1,2^. Derangement of this migratory activity has been reported to cause abnormalities of pubertal development, including idiopathic hypogonadotropic hypogonadism (IHH) and self-limited delayed puberty^3–5^. In IHH, patients cannot complete pubertal development and they need long-term sex hormone replacement, whereas self-limited delayed puberty patients do enter puberty spontaneously although later than in the normal population^6^.

Whilst the cut-off for the age of puberty onset in healthy individuals is variable, for example amongst different ethnic groups and between the developed and developing world, traditionally delayed puberty (DP) is diagnosed when puberty has not begun at the age of 13.5 years in boys and 13 years in girls^7^. Although DP can be caused by a variety of underlying aetiologies, the most common cause is self-limited delayed puberty, also known as constitutional delayed growth and puberty, accounting for 63–82% of boys and 30–56% of girls with DP from large cohort studies^8–10^. Between half and three-quarters of individuals with self-limited delayed puberty have a family history of late puberty, most commonly with an autosomal dominant inheritance pattern, with or without complete penetrance^11^. Several underlying mechanisms have been identified which lead to self-limited delayed puberty, including defects of GnRH neuronal development^5,12^, maturation and function^13^, upstream controllers of the HPG axis^14^ and regulators of energy homeostasis^15^.

Abnormal GnRH neuronal migration during fetal development appears to be a crucial underlying mechanism, with mutations in several genes coding for migration guidance proteins, including *IGSF10*^5^, *HS6ST1*^16^, *FEZF1*^17^, and *PROKR2*^4^ resulting in either abnormal pubertal timing or GnRH deficiency. While severe loss-of-function defects in genes coding for GnRH migration factors are known to cause GnRH deficiency (i.e., IHH), we hypothesized that a milder burden of mutation in the same genes, including monoallelic rather than biallelic or hypomorphic rather than loss-of-function mutations, might not cause GnRH deficiency but affect the timing of pubertal onset. Based on this hypothesis, we explored whether genetic controllers of pubertal timing can be discovered by genomic analysis in familial self-limited delayed puberty, by focusing on genes in pathways related to development of the GnRH network previously reported to cause IHH.

## Results

### Exome sequencing of families with self-limited delayed puberty identifies variants in *Coiled-Coil Domain Containing 141 (CCDC141)*

**Fig. 1:**
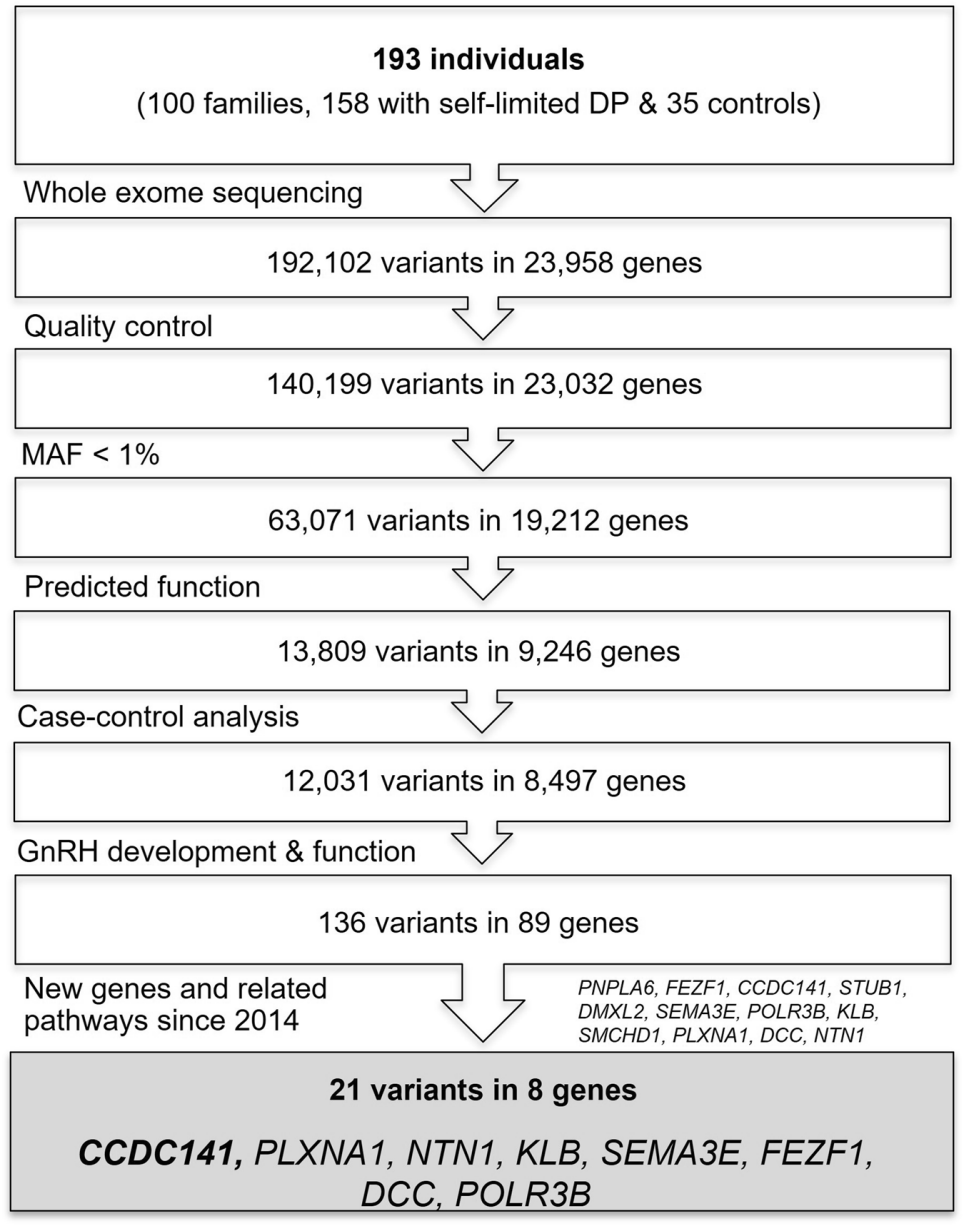
Whole exome sequencing analysis pipeline. Analytic pipeline for genetic analysis in self-limited delayed puberty pedigrees, focusing on genes reported in the literature to cause IHH since 2014^18,36^.

Whole exome sequencing (WES) data from a large Finnish cohort consisting of 100 DP families was analysed, with sequencing data from a total of 193 individuals. Initially, 192,105 variants in 23,958 genes were identified from this dataset. Variants were filtered for rare, predicted deleterious variants, in 12 recently described candidate genes with potential biological relevance. Twenty-one predicted deleterious variants in eight genes, recently reported to regulate GnRH neuronal development, were identified in the probands and their relatives with self-limited DP^18^ (Fig. 1). *CCDC141* (NM_173648, synonyms: “Coiled-coil protein Associated with Myosin II and DISC1”, or “*CAMDI*”) was found to have a high prevalence of predicted deleterious variants in our cohort. These were identified in 6% of self-limited delayed puberty probands, with a variant identified in only one unaffected parent of one of these probands and not found in other unaffected relatives sequenced (*n* = 35). As an additional filtering step to prioritize candidates for characterization, we analysed the WES data returned from our cohort by whole gene rare variant burden testing for all rare (minor allele frequency (MAF) < 1%) and predicted deleterious variants in the gnomAD database. Rare, predicted deleterious variants in *CCDC141* appeared enriched in self-limited delayed puberty subjects as compared to the Finnish control population, although this was not significant after FDR correction (*gnomAD analysis unadjusted p-value* = *0.05; FDR adjusted p value* = *0.21*).

*CCDC141* encodes a 1530 amino acid protein which has been reported to be expressed in migrating GnRH neurons and olfactory axons between embryonic day (E) 11.5 and E14.5 in mice^19,20^. Knockdown of this gene in embryonic mice has been shown to impair cortical neuronal migration with disoriented centrosomes in a myosin II-dependent manner^19^. Thereafter, homozygous mutations of *CCDC141*, or heterozygous mutations in combination with mutations in other known IHH genes, were reported in Kallmann syndrome and IHH patients, although *CCDC141* mutations have never previously been reported in familial self-limited delayed puberty^20,21^. These studies also showed that knockdown of *Ccdc141* in mice nasal explants resulted in impaired GnRH neuronal migration without olfactory axon abnormalities^20,21^.

### Rare, predicted deleterious variants in *CCDC141* found in six families with self-limited delayed puberty

**Fig. 2:**
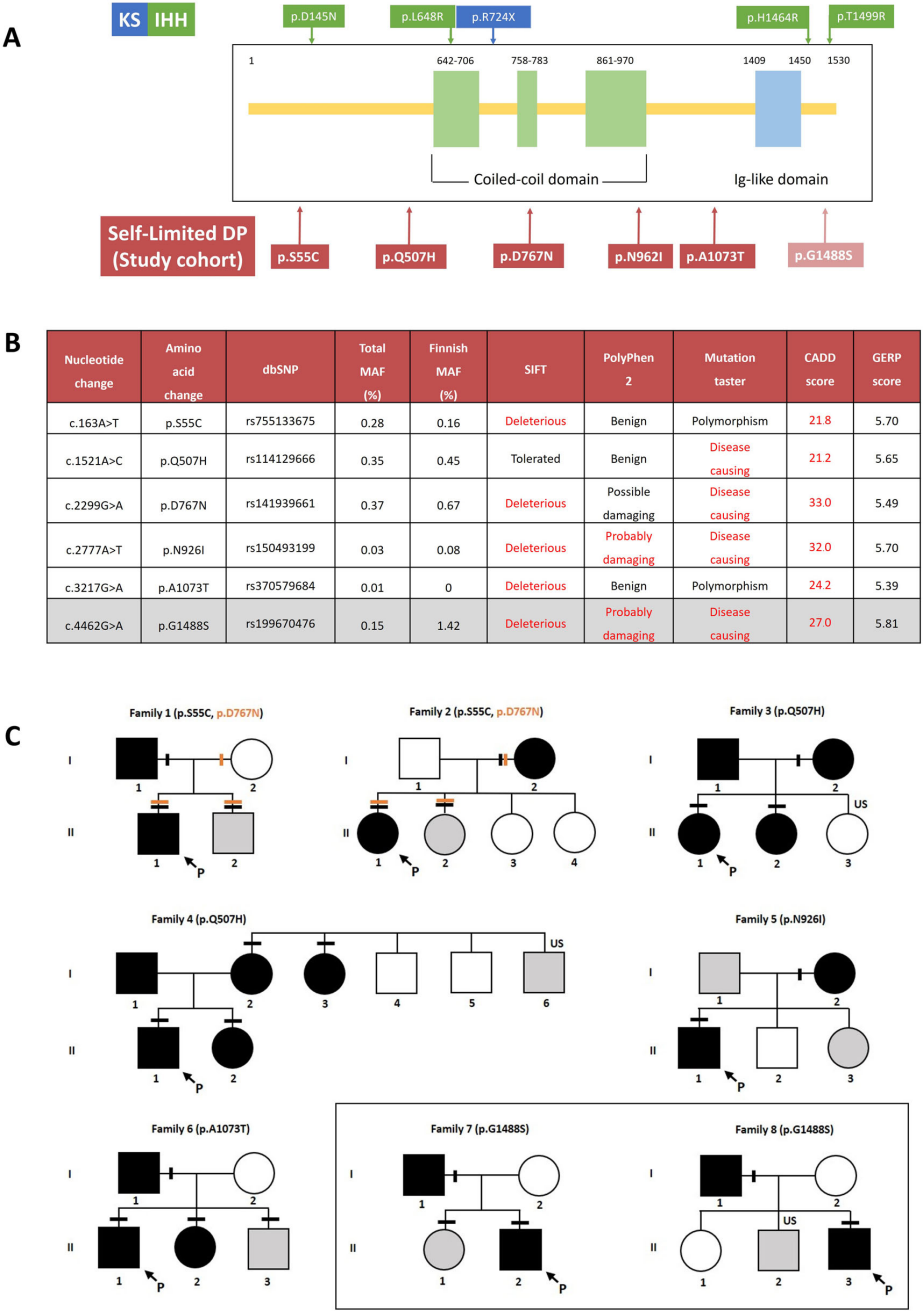
Identified *CCDC141* variants from WES in our self-limited delayed puberty cohort. **A** Structure of CCDC141 protein and location of all variants found from WES data. Numbers correspond to amino acid position. IHH—mutations in idiopathic hypogonadotropic hypogonadism patients described in the literature^21^, KS—mutations in Kallmann syndrome patients described in the literature^20^. **B** MAF in the total and ethnic-specific populations from the gnomAD database, in silico prediction of *CCDC141* variants according to web-based prediction software programs (with red font indicating a deleterious or damaging prediction), and GERP score indicating conservation of genetic sequences across species. Variant p.Gly1488Ser has MAF > 1 in Finnish gnomAD population, thus shown shaded in gray. **C** Pedigrees of families identified with *CCDC141* variants. Squares indicate males, circles females. Black symbols represent DP phenotype, gray represents an unknown phenotype (either too young at time of phenotyping or insufficient data available), clear symbols represent unaffected individuals. The arrow with “P” indicates the proband in each family and “US” indicates un-sequenced due to lack of DNA. The mutation in each family is given next to the family number; a horizontal bar (black or orange) above an individual’s symbol indicates they are heterozygous for that mutation as confirmed by WES and verified by Sanger sequencing. Pedigrees carrying variant p.Gly1488Ser shown apart as MAF > 1 in Finnish gnomAD population.

Five predicted deleterious variants of *CCDC141* were identified in the WES dataset of our cohort. The variants are located along the whole length of the gene, all at different positions to those reported from previous studies (Fig. 2A)^20,21^. Two variants lie within the coiled-coils [NC_000002.12 (NM_173648): c.2299G>A: p.Asp767Asn and NC_000002.12 (NM_173648): c.2777A>T: p.Asn926Ile] while three lie in non-annotated regions of CCDC141 [NC_000002.12 (NM_173648): c.163A>T: p.Ser55Cys, NC_000002.12 (NM_173648): c.1521A>C: p.Gln507His, and NC_000002.12 (NM_173648): c.3217G>A: p.Ala1073Thr. All variants had a MAF of less than 1% of the total population in the gnomAD database (accessed Aug 2021) (Fig. 2B)^22^. A sixth variant [NC_000002.12 (NM_173648): c.4462G>A: p.Gly1488Ser] was identified above the allele frequency threshold with a MAF of 1.4% in the Finnish gnomAD population. All *CCDC141* variants are heterozygous missense variants that affect amino acids that are highly conserved among homologs as indicated by Genomic Evolutionary Rate Profiling (GERP) score (Fig. 2B).

### Families with *CCDC141* variants display autosomal dominant inheritance and classical self-limited delayed puberty

Twenty-one individuals in six families were identified from WES data to carry predicted deleterious variants in *CCDC141* (Fig. 2C). In the majority of these six pedigrees, there was clear segregation of the variants with the self-limited delayed puberty trait. However, in two families there was a complex inheritance pattern with compound heterozygosity in one pedigree (Family 1) and inheritance of two variants on the same allele in a further family (Family 2). Individual I.2 in family 1 carried the p.Asp767Asn variant, as identified by Sanger sequencing, but did not manifest a phenotype of DP. In addition, there were 2 families that showed a bilineal inheritance pattern (Family 3 and 4; p.Gln507His), in whom the father also had DP; but despite WES analysis the genetic defect leading to this individual’s phenotype of DP remains unknown. None of the individuals from these pedigrees carried predicted deleterious variants in other genes known to cause self-limited delayed puberty or IHH.

The probands from these families had typical clinical and biochemical features of self-limited delayed puberty, with delayed onset of Tanner stage G2 and delayed peak height velocity. They displayed low gonadotropin concentrations with low or undetectable sex steroid concentrations and delayed bone age at presentation. All had normosmia by self-report. After completion of follow up, all were found to have spontaneously entered puberty before the age of 18 years without sex steroid treatment (Table 1). None of the individuals carrying *CCDC141* predicted deleterious variants displayed syndromic or psychiatric disorders. The pedigrees of the two families in which the p.Gly1488Ser variant was identified are included for interest (Fig. 2C), as prediction tools indicated this variant to be deleterious, although the MAF in the Finnish gnomAD population is 1.4%.

**Table 1:** Clinical data of probands carrying *CCDC141* variants.

Family number	1	2	3	4	5	6	7	8
Identified CCDC141 variants	p.Ser55Cys p.D767N	p.Ser55Cys p.Asp767Asn	p.Gln507His	p.Gln507His	p.Asn926Ile	p.Ala1073Thr	p.Gly1488Ser	p.Gly1488Ser
Clinical data at 1st assessment								
Sex	M	F	F	M	M	M	M	M
Age (year)	15.1	13.6	14.4	15.2	15.4	14.9	12	13.97
Bone age (year)*	–	12	10.5	14	11.5	13	7.5	12.75
TV (mls)	3	3	–	4	1	4	2	3
PH Tanner stage	1	1	1	1	1	1	1	1
G/B Tanner stage	1	1	1	2	1	2	1	2
BMI (kg/m^2^)	17.6	25	17.6	21.2	19.9	20.5	–	21.5
LH (IU/L)** (0.1–0.6)***	0.9	0.2	0.1	0.2	0.4	1.1	–	0.3
FSH (IU/L)** (0.1–0.9)***	1.0	0.4	0.2	0.4	0.9	2.1	–	0.8
T (nmol/L)** (0.1–1.0)***	1.1	0.9	–	0.3	1.0	1.4	–	0.3
E2 (pmol/L)** (<8)***	–	–	<20	–	–	–	–	–
Age at secondary sexual characteristics (year)								
G2/B2	15.3	14.5	14.7	15.2	15.4	14.7	15.7^	14.3
Take-off	14.6	14.6	14.7	15.5	15.4	14.2		14.3
PHV	–	16.1	15.4	15.9	15.9	–	15.0	14.9
Induction of puberty								
Yes/No	No	No	No	No	No	No	No	No

*TV* testicular volume in mL, *PH* pubic hair, *G* genital, *B* breast stage, *IU* international units, *G2* genital stage 2, *B2* breast stage 2, *PHV* peak height velocity.

* Bone age estimated by the Greulich and Pyle method.

**Baseline values

***Normal ranges for prepubertal concentrations given in parenthesis.

^Estimated age as G1 to G3 transition occurred between clinic visits. Data for probands carrying the p.Gly1488Ser variant is shaded gray in view of the Finnish gnomAD MAF for this variant being above the 1% threshold.

### In silico analysis predicts CCDC141 variants found in self-limited delayed puberty pedigrees to be deleterious

No experimental structure of CCDC141 was available at the time of the study and therefore a 3D model structure was generated (confidence score >98%, which indicates that protein folding and the core of the protein were modeled accurately) (Fig. S1A). A sequence-structure analysis showed the following results. For variants p.Ser55Cys and p.Gln507His the modeling did not predict structural damage; however, in p.Ser55Cys the conserved polar hydrophilic serine residue on the protein surface is replaced with a hydrophobic cysteine and in p.Gln507His the neutrally charged glutamine is substituted with the positively charged histidine. Variants p.Asp767Asn, p.Asn926Ile, and p.Ala1073Thr were predicted to be damaging. In p.Asp767Asn the invariant negatively charged aspartic acid is replaced with a non-charged residue. p.Asn767 is part of a large charged area, possibly representing a protein interaction site and such a substitution would disrupt this functional site (Fig. S1B). In p.Asn926Ile, the invariant polar, non-charged asparagine which may form stabilizing hydrogen bonds, is replaced with the hydrophobic isoleucine. In p.Ala1073Thr the invariant hydrophobic residue occupied by alanine is replaced with the polar threonine. Position 1488 was not covered by the 3D model. However, the change from glycine to serine in the p.Gly1488Ser variant was also predicted to be deleterious by all four in silico tools (Fig. 2B).

### CCDC141 mutants display an abnormal subcellular distribution

CCDC141 has a crucial role in cell migration through the positioning and orientation of the centrosome^19^. To facilitate cell migration, the centrosome needs to be moved forward into the cell protrusion segment where it is fixed before subsequent nuclear movement occurs^23^. Thus, centrosome stabilization, which is facilitated by pericentriolar materials including acetylated (Ac)-tubulin, is a crucial step for cell migration^24^. CCDC141 can enhance α-tubulin acetylation in the centrosome, by inhibiting the activity of HDAC6, a deacetylase enzyme of α-tubulin. This evidence was found via analysis of *Ccdc141*-knockdown mice which showed abnormal cortical neuronal migration and centrosomal disorientation^19^, in addition to decreased Ac-tubulin accumulation being observed in the centrosomal area of *Ccdc141*-knockout brain lysates^25^.

**Fig. 3:**
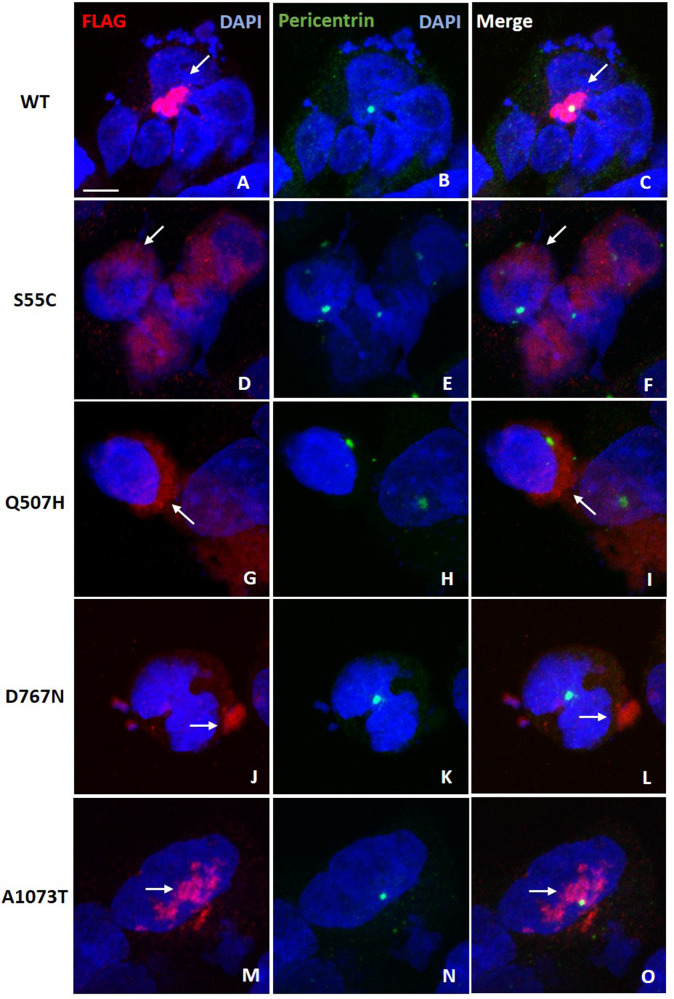
Abnormal subcellular localization of CCDC141 mutant proteins. Immunofluorescence was carried out in HEK293T cells with transient expression of CCDC141, WT (**A**–**C**), p.Ser55Cys (**D**–**F**), p.Gln507His (**G**–**I**), p.Asp767Asn (**J**–**L**), and p.Ala1073Thr (**M**–**O**). CCDC141 was stained by anti-FLAG (Red), as indicated by the white arrows. Centrosome was marked by anti-pericentrin (Green). WT wildtype. Scale bar in **A** (applies to all), 10 µm.

We therefore sought to characterize the impact of our identified mutations on the location and function of the CCDC141 protein. Whilst we found no difference in total CCDC141 protein expression between WT and variant-expressing HEK293T cells (Fig. S2), WT CCDC141 subcellular localization was found to be tightly associated with the centrosome (Fig. 3A–C), as previously reported^19^. However, a more diffusely scattered pattern of localization was observed for p.Asp767Asn and p.Ala1073Thr mutants (Fig. 3J–O), and p.Ser55Cys and p.Gln507His mutant proteins were widely distributed throughout the cytoplasm (Fig. 3D–I). The other two predicted deleterious variants (Asn926Ile and Gly1488Ser) could not be successfully studied in vitro due to technical difficulties with site-directed mutagenesis.

### Mutations in *CCDC141* affect the distribution of acetylated tubulin

**Fig. 4:**
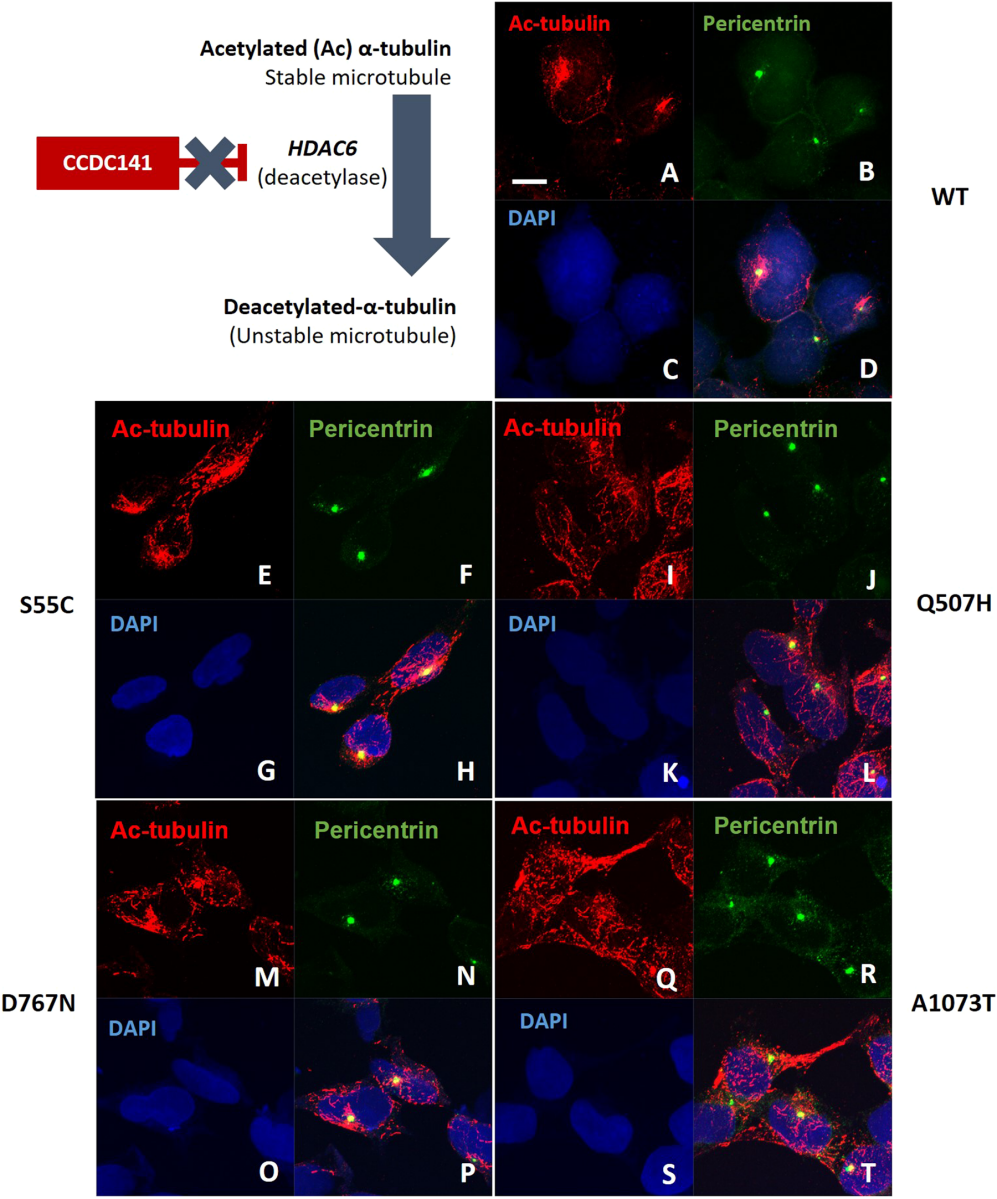
Abnormal Ac-tubulin distribution in CCDC141 variants-expressing cells. The location and accumulation of Ac-tubulin was assessed by immunofluorescence staining of Ac-tubulin (Red) in HEK293 cell lines with stable expression of *CCDC141*, WT (**A**–**D**), p.Ser55Cys (**E**–**H**), p.Gln507His (**I**–**L**), p.Asp767Asn (**M**–**P**), and p.Ala1073Thr (**Q**–**T**). The Centrosomal area was marked by pericentrin (Green) and nucleus was marked by DAPI (Blue). Scale bar in **A** (applies to all), 10 µm.

As discussed above, CCDC141 has a role in the conversion of acetylated to deacetylated α-tubulin in centrosomes^21^ (Fig. 4). Therefore, we studied the expression and distribution of Ac-tubulin in the presence of WT and mutant CCDC141. We could not observe any difference in Ac-tubulin expression in total cell lysates between WT and all mutant CCDC141 proteins (Fig. S3). However, in parallel with the perturbed subcellular localization of mutant CCDC141, Ac-tubulin was found to have an abnormal distribution in variant-expressing HEK293 cells. In contrast to the predominantly pericentrin-associated localization of Ac-tubulin in cells expressing WT CCDC141 (Fig. 4A–D), Ac-tubulin was dispersed through the cytoplasm when in the presence of mutant CCDC141 (Fig. 4E–T). As CCDC141 is thought to mediate its role in deacetylation via HDAC6, we also assessed HDAC6 activity in these cells (Fig. S4), but no significant difference in enzymatic activity of HDAC6 was observed with the presence of mutant CCDC141.

### Mutations in *CCDC141* affect cell migration

*CCDC141* knockdown has been shown to result in abnormal cortical or GnRH neuronal cell migration^19–21,25^, and recently *CCDC141* mutations have been reported in patients with KS and IHH^20,21^. In view of this, and the above evidence of impaired centrosomal localization of CCDC141 and Ac-tubulin in the presence of our identified deleterious variants, we investigated the migratory properties of HEK293 cells stably expressing either WT or these CCDC141 variants via a scratch assay. Whilst the migratory GnRH neuron-like cell line GN11 would have been our preferred option for this migratory assay, the transfection of these large CCDC141 plasmids into this cell line was technically not possible, thus rendering this option unavailable.

**Fig. 5:**
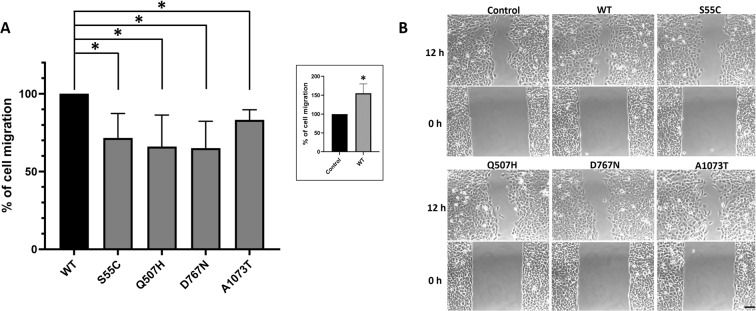
Variants of CCDC141 identified in patients with self-limited delayed puberty result in impaired cell migration. Scratch assay was carried out in *CCDC141*-overexpressing HEK293 cells. A percentage of cell migration (wound closure) at 12 h in mutant-transfected vs. WT-transfected HEK293 cells (*n* = 3). The inset to **A** shows the percentage of cell migration of WT cells, compared to that of non-transfected cells as control (100%). Error bars show SD. **B** Representative contrast light microscope pictures at 0 and 12 h. Scale bar, 100 µm, *p < 0.05.

HEK293 cells stably expressing CCDC141 variants displayed significantly reduced migration, with wound closure, as compared with WT, at 71.6 ± 9.0% for variant p.Ser55Cys (*p* = 0.04), 66.1 ± 11.7% for p.Gln507His (*p* = 0.04), 65.1 ± 9.9% for p.Asp767Asn (*p* = 0.02), and 83.2 ± 3.7% for p.Ala1073Thr (*p* = 0.01) (Fig. 5A, B). In contrast, cells overexpressing WT *CCDC141* had an increased rate of cell migration as compared to untransfected cells in which endogenous CCDC141 is present, supporting previous evidence of the role of *CCDC141* in cell migration. No obvious morphological differences were observed between WT and mutant expressing cells and levels of cell proliferation were equal (Fig. S5), supporting that the delayed wound closure seen with mutant expressing cells was due to impaired migratory properties.

## Discussion

Self-limited delayed puberty is the most common cause of pubertal delay in adolescence, but its pathogenesis remains poorly understood. This condition is known to cluster in families, most commonly with an autosomal dominant inheritance pattern^11^. To date, only a small number of underlying genetic regulators of self-limited delayed puberty have been identified and most of them are related to GnRH biology, including GnRH neuronal migration, maturation and function^5,12,14^. Furthermore, the underlying mechanisms by which defects in these genes impair GnRH neuronal development has been minimally described. Genetic analyses in IHH families show that self-limited delayed puberty and IHH may share some overlap of their pathophysiology, with cases described of homozygous mutations in genes such as *GNRHR*^26,27^ and *TAC3* and its receptor^27^ causing IHH, whilst heterozygous carriage of the same variants is associated with the phenotype of self-limited delayed puberty^16,26,27^.

In this study, we analysed WES data from patients with self-limited delayed puberty from our large familial cohort, to determine if pathways related to development of the GnRH network, previously reported to contribute to IHH, also underlie this condition. Recently, predicted deleterious variants in *CCDC141* have been identified in patients with KS and IHH, but *CCDC141* has not been previously analysed in individuals with self-limited delayed puberty. We identified six probands who carried altogether five different predicted deleterious variants in *CCDC141*, accounting for 6% of the cohort. A sixth predicted deleterious variant (p.Gly1488Ser) was found in two further families, but the relevance of this variant remains unclear as the allele frequency in the Finnish population is 1.4%. None of the variants were previously reported in patients with IHH or KS, and all variants were heterozygous without mutations in other known IHH genes, in contrast to the homozygous and digenic or oligogenic carriage observed in patients with GnRH deficiency^20,21^.

CCDC141 is a protein involved in the development of the cortical architecture of the central nervous system. Expression of this gene has been shown in migrating GnRH neurons and olfactory axons between E11.5 and E14.5^20^. CCDC141 has a crucial role in neuronal migration in both cortical and GnRH neurons^19–21,25^. Whole gene knockdown of *Ccdc141* in mice nasal explants was shown to impair GnRH neuronal migration and is likely to be responsible for the phenotype of KS and IHH in human^20,21^; but these studies have not characterized in vitro specific variants in this gene.

Based on these studies, we aimed to characterize the functional consequence of variants identified in individuals with self-limited delayed puberty. In keeping with a model where deficiency of CCDC141 impairs cell migration, previous studies have reported that CCDC141 has a role in centrosome position, orientation, and stabilization during the migratory process^19^. Thus, the presence of CCDC141 at the centrosome is likely to be essential for its function, with mis-localization leading to abnormal cell migration. Our study demonstrated that cells expressing variants identified in individuals with self-limited delayed puberty indeed had abnormal subcellular localization of these CCDC141 proteins.

Additionally, CCDC141 has been shown to have a role in deacetylation of α-tubulin in the centrosome^19,25^. Centrosomal fraction of brain lysates from *Ccdc141* knockout mice showed an impaired expression of Ac-tubulin, which is crucial for centrosomal stability and microtubule assembly^25^. Our study found that the expression of variant CCDC141 also affected Ac-tubulin distribution, which instead of displaying its normal pericentrosomal location, was dispersed through the cytoplasm. We proposed that the reduction of Ac-tubulin in the centrosomal area would result in centrosomal instability and thus impair cell migration. In keeping with this, each variant tested in an assay of cell migration was shown to significantly reduce migratory capacity. Finally, the effects of mutant CCDC141 may have been mediated via reduced inhibition of HDAC6 enzymatic activity, leading to a reduction of acetylated tubulin, but this reduction was not significant in HEK293T cells.

An intact GnRH network is necessary for the appropriate pacing of pubertal onset, as demonstrated by human diseases and animal models^28,29^. The presence of GnRH neurons in the hypothalamus at the correct developmental time seems to be an essential factor to enable GnRH neurons to connect to adjacent neuronal and glial cells to establish a functional network. Failure of CCDC141 function, and its permissive function on Ac-tubulin-mediated centrosome stabilization, would thus lead to abnormal cell migration, impacting on the development of the GnRH neuroendocrine network. Impairment of the neuroendocrine responses of such a network would result in an increased “threshold” for the onset of puberty, with an ensuing delay in pubertal timing^5^. Thus, misplaced or delayed arrival of GnRH neurons to the hypothalamus, leading to malformation of the GnRH neuroendocrine network at the required developmental window^30,31^, may result in a spectrum of phenotypes from self-limited delayed puberty to IHH depending on the severity of the defect. This would parallel the results previously found in patients carrying heterozygous mutations of *IGSF10* who manifest a phenotype of self-limited delayed puberty, where knockdown of this gene led to abnormal GnRH migration in cell and animal models^5^.

In summary, we have identified five deleterious variants in *CCDC141* in families with self-limited delayed puberty, four of which have been demonstrated to result in delayed cell migration in a cellular model. These findings reinforce the importance of GnRH neuronal migration during fetal development in influencing the timing of pubertal onset, and suggest that disruption of this process can underlie both the conditions of self-limited delayed puberty and IHH.

## Methods

### Patients

Subjects of this study were included from a large Finnish cohort of families with DP which has been described previously^11^. In brief, the subjects referred to pediatric care centers in central and southern Finland from 1982 to 2004 were included in this cohort using accurate diagnostic methodology, with objective evidence from physical maturation and precise and centralized growth charts rather than historical self-recall. The subjects who met the diagnostic criteria for delayed puberty, defined as the onset of Tanner stage G2 (testicular volume >3 ml) at >13.5 year in boys or Tanner stage B2 at >13.0 year in girls (i.e., two SD later than average pubertal development) were recruited (*n* = 492). To exclude chronic illness as a cause for DP, medical history, carefully physical examination, and routine laboratory investigations were performed. IHH, if suspected, was excluded by spontaneous pubertal development at follow-up. Half of the patients in this cohort were treated by exogenous sex steroid for pubertal induction. Their follow-up was completed when the patients achieved full pubertal development (Tanner stage >G4 or >B4).

Relatives of DP probands were invited to take part in the cohort. Medical history and pubertal timing information were obtained from previous growth records and structured interviews. The criteria for DP in probands’ family members were one of three of the following: (1) age at take-off or (2) peak height velocity (PHV) occurring 1.5 SD beyond the mean, i.e., age at take-off exceeding 12.9 and 11.3 years, or age at PHV exceeding 14.8 and 12.8 years in males and females, or 3) age at attaining adult height more than 18 or 16 years, in males and females, respectively. All family members were assigned a clinical status of affected, unaffected, or unknown. Those with unknown status were either too young to diagnose or had insufficient growth data available.

### Study approval

Human patients: written informed consent was obtained from all participants. The study protocol was approved by the Ethics Committee for Pediatrics, Adolescent Medicine and Psychiatry, Hospital District of Helsinki and Uusimaa (570/E7/2003). UK ethical approval was granted by the London-Chelsea NRES committee (13/LO/0257). The study was conducted in accordance with the guidelines of The Declaration of Helsinki.

### DNA sequencing and variant analysis

WES was performed on DNA extracted from peripheral blood leukocytes of 193 individuals: 100 probands, 58 affected, and 35 unaffected family members. WES samples were prepared using either Nimblegen V2 or Agilent V4 platforms then subjected to massively parallel sequencing on the Illumina HiSeq 2000. The exome sequences were aligned to the UCSC hg19 reference genome using Burrows-Wheeler Alignment tool (BWA-MEM [bwa-0.7.12]) and Picard Tools software (version 1.119). The Genome Analysis Toolkit (GATK-3.4–46) was used to mark PCR duplicates, realign around indels, recalibrate quality scores, and call variants using the HaplotypeCaller algorithm.

Potentially pathogenic variants were identified by filtering for quality control, minor allele frequency, predicted functional annotation, case–control analysis (segregation with trait and identification of variants in multiple families), followed by biological relevance (Fig. 1). Quality control included thresholds for call quality, read depth, and Phred strand bias. Variants called with a genotype quality (GQ) < 20 and read depth less than 20 were excluded. Only variants with MAF < 1% across total or ethnic-specific populations in the 1000 Genomes database, the NHLBI exome variant server, and ExAC/Genome Aggregation Database (gnomAD) databases were prioritized. Thereafter, predicted functional annotation involved prioritizing nonsense, exonic missense, splice site variants, structural or promoter changes, or variants deleterious to a microRNA. The identified missense variants were assessed to be deleterious by prediction software tools: sorting intolerant from tolerant (SIFT)^32^, polymorphism phenotying v2 (PolyPhen2)^33^, mutation taster^34^, and combined annotation dependent depletion (CADD) score^35^, with a threshold of ≥2 of four annotation tools predicting pathogenicity. Case–control analysis refers to retaining only those genes with variants present in ≥*n* − 1 affected individuals (where *n* = number of affected individuals in a given pedigree), not present in more than one of 35 unaffected members from 18 cohort families (eight families shown in Fig. 2, and ten further families from this cohort). Thereafter, filters for biological relevance were applied: firstly, retaining variants in genes related to GnRH neuronal migration and development and secondly, in genes reported in the literature to be associated with conditions of gonadotropin deficiency published since January 2014. This resulted in a set of 12 genes used to filter the WES data as a virtual panel^18,36^.

Results were verified, in parallel, by applying statistical thresholds for enrichment of rare, predicted deleterious variants in our cohort via whole gene rare variant burden testing with FDR multiple comparison adjustment for 12 selected candidate genes^37^. Fisher’s exact test was used to compare the prevalence of rare (total population minor allele frequency less than 1%) and deleterious (as predicted by SIFT and Polyphen-2 prediction tools) variants identified by WES in our cohort, with the Finnish population using the Genome Aggregation Database (gnomAD) data (accessed October 2021), Supplementary Table 1. An assumption of 25,120 alleles sequenced for all variants was made, which may underestimate the frequency of variants in controls and may skew the results of burden testing. Potentially pathologic variants identified using NGS were confirmed using Sanger sequencing and no discrepancy between these results was detected. All available family members that had not undergone NGS were Sanger sequenced for the family variant to assess segregation within pedigrees.

### In silico analysis

The CCDC141 amino acid sequence, corresponding to transcription of 1530 amino acid (Ensembl Id NP_775919.3), was retrieved from Uniprot (UniProt ID Q6ZP82–2 and E7ERF0). The amino acid sequences for CCDC141 orthologues were also retrieved from Uniprot.

A structural model of CCDC141 was generated using the Phyre2 prediction program^38^ and results were compared to those obtained using two additional state-of-the-art methods: SwissModel^39^ and I-Tasser^40^. For the structural analysis of missense variants, the following structural elements were considered in the wild-type and variants CCDC141 structure: steric clash, hydrogen bond breakage, salt bridge breakage, cavity alteration, disulfide bond breakage, disallowed phi/psi angles and secondary structure change, as detailed in Ittisoponpisan et al.^41^. The CCDC141 electrostatic potential was calculated using the PBEQ program^42^, which computes the protein electrostatic potential by solving the Poisson–Boltzmann equation. Because of the low sequence identity (<30%) between CCDC141 and the templates used for homology modeling, accurate atom-based analysis on the possible structural impact of CCDC141 missense variants could not be performed.

### Constructs and protein expression

A vector containing the full length (NM_173648) cDNA of human *CCDC141-FLAG* was obtained from GenScript. *CCDC141* mutation-carrying vectors were generated by using QuikChange II Site-Directed Mutagenesis Kit (Agilent Technologies) following the company protocol, by which four of six variants were successfully generated and taken forward for in vitro studies. Primer sequences are given in Supplementary Tables 2 and 3.

### Cell culture

Human embryonic kidney (HEK) 293T cells and HEK293 cells (sourced from ATCC) were used for transient and stable transfection, respectively. Cells were cultured in Dulbecco’s Modified Eagle’s Medium (DMEM)—high glucose (Sigma-Aldrich) supplemented with 10% fetal bovine serum (Invitrogen) and 1% penicillin–streptomycin (Sigma-Aldrich) in 5% CO_2_ at 37 °C. Cells were checked for mycoplasma contamination (MycoAlert Detection Kit, Lonza) on a monthly basis and were contamination-free. Vectors were transfected into HEK293T or HEK293 cells using Lipofectamine 2000 (Invitrogen) according to the manufacturers’ instructions. Transfected HEK293 cells were selected with G418 for generating stably-expressing CCDC141 cell lines (1 mg/ml, Sigma-Aldrich).

### Western blot

Total proteins were extracted from cells with RIPA lysis buffer (Sigma-Aldrich) supplemented with protease inhibitor cocktail (Sigma-Aldrich), and 20–30 μg of proteins were size-separated using NuPage BisTris gels 4–12% (Invitrogen) and transferred to nitrocellulose membranes (Promega). Membranes were blocked with PBS containing 0.1% Tween-20 and 5% non-fat dry milk and incubated with antibodies to CCDC141 (1:1000 dilution, SAB3500670, Sigma-Aldrich), Ac-tubulin (1:1000 dilution, ab179484, Abcam), Proliferating Cell Nuclear Antigen (PCNA 1:2000 dilution, 2586, Cell Signaling Technologies) and Glyceraldehyde-3-Phosphate Dehydrogenase (GAPDH 1:10,000 dilution, G9545, Sigma-Aldrich and MA5-15738, Thermofisher). Anti-rabbit and mouse IRDye680 or IRDye800 (Licor) were used as secondary antibodies (1:10,000 dilution). Immunoblots were scanned with the Odyssey® Fc Imaging System (Licor).

### Migration assay

A scratch assay method was used to assess migration of HEK293 cells stably expressing either wild-type (WT) or four key deleterious variants of CCDC141 by using Culture-Insert 2 Well in µ-Dish 35 mm (Ibidi). The procedure was carried out following the company’s instructions. Briefly, 70 µL aliquots of 7 × 10^5^ cells in suspension/1 mL in DMEM were applied into each well. After 24 h, the silicone block was removed by sterile tweezers, cells were washed and then incubated with 2 mL of culture medium. Pictures from four different regions were taken at 0 and 12 h and analysed by measuring the area of cell migration with the TScratch program.

### Immunofluorescence

HEK293T cells were seeded on cover slips and transiently transfected with either WT or CCDC141 variants using Lipofectamine, and after 48 h were utilized for immunofluorescence. HEK293 cells stably expressing either WT or *CCDC141* variants were used for immunofluorescence 24 h after seeding. Cells were fixed in 4% paraformaldehyde for 15 min, then were permeabilised with 0.1% Triton X-100 in PBS for 30 min and blocked with blocking buffer [10% bovine serum albumin (BSA) in PBS] for 30 min. Anti-FLAG (dilution 1:1000; F1804, Sigma-Aldrich), anti-Ac-tubulin (dilution 1:500; ab179484, Abcam), and anti-pericentrin (dilution 1:2000; ab4448, Abcam) diluted in 3% BSA/PBS were used to incubate cells for 2 h at RT. Secondary Ab, anti-Mouse IgG-FITC (Invitrogen), at a 1:1000 dilution in 3% BSA/PBS was applied for incubation for 45 m at RT. Nuclei were stained with 4′,6-diamidino-2-phenylindole (DAPI) by using Vectorshield mounting medium with DAPI (Vector laboratories). Images were taken by a LSM 510 laser scanning confocal microscope (Zeiss) and processed using Adobe Photoshop CS6.

### HDAC6 activity assay

Total cell lysates from CCDC141-expressing HEK293 cells were analysed with a HDAC6 activity assay (Biovision) (*n* = 3). HEK293 cells were transiently transfected with either *CCDC141* WT or mutants with Lipofectamine 2000 (Invitrogen) and were incubated for 48 h before cells were lysed. Assay analysis was carried out following the company’s instructions. The fluorescence read-out was measured at Ex/EM 380/490 nm in an endpoint mode at 37 °C using FLUOstar Omega Multimode Microplate Reader (BMG Labtech).

### Statistical analysis

Whole gene rare variant burden testing was performed post sequencing. Fisher’s exact test was used to compare the prevalence of deleterious variants in our cohort with the Finnish population, using the gnomAD Browser, Cambridge, MA: accessed September 2020), with a multiple comparison adjustment applied post hoc using the Benjamini & Hochberg method^37^.

Scratch assay and HDAC activity results are presented as percentage of WT (mean ± SEM). The comparison between two variables was calculated by unpaired *t*-test (two-tail). *P*-values less than 0.05 were considered statistically significant. The statistical analysis was performed using GraphPad Prism 8 (GraphPad Software).

### Reporting summary

Further information on research design is available in the Nature Research Reporting Summary linked to this article.

## Supplementary information

Reporting Summary

Supplementary Information

## Data Availability

The WES datasets generated during and/or analysed during the current study are available in Sequence Read Archive under accession number PRJNA779391. The other materials generated from this study are available from the corresponding author on reasonable request.
