# Supplementary material for: Whole exome sequencing identifies deleterious rare variants in CCDC141 in familial self-limited delayed puberty
Source: NPJ Genom Med. 2021 Dec 20;6:107. doi: 10.1038/s41525-021-00274-w (PMC8688425; doi:10.1038/s41525-021-00274-w)
Supplement: Supplementary file 1 — Reporting Summary [file 41525_2021_274_MOESM1_ESM.pdf]

nature portfolio

Corresponding author(s): Dr Sasha Howard

Last updated by author(s): Jun 14, 2021

Reporting Summary

Nature Portfolio wishes to improve the reproducibility of the work that we publish. This form provides structure for consistency and transparency in reporting. For further information on Nature Portfolio policies, see our Editorial Policies and the Editorial Policy Checklist.

Statistics

For all statistical analyses, confirm that the following items are present in the figure legend, table legend, main text, or Methods section.

n/a	Confirmed	
☐	☒	The exact sample size (n) for each experimental group/condition, given as a discrete number and unit of measurement
☐	☒	A statement on whether measurements were taken from distinct samples or whether the same sample was measured repeatedly
☐	☒	The statistical test(s) used AND whether they are one- or two-sided<br>*Only common tests should be described solely by name; describe more complex techniques in the Methods section.*
☒	☐	A description of all covariates tested
☐	☒	A description of any assumptions or corrections, such as tests of normality and adjustment for multiple comparisons
☐	☒	A full description of the statistical parameters including central tendency (e.g. means) or other basic estimates (e.g. regression coefficient) AND variation (e.g. standard deviation) or associated estimates of uncertainty (e.g. confidence intervals)
☒	☐	For null hypothesis testing, the test statistic (e.g. F, t, r) with confidence intervals, effect sizes, degrees of freedom and P value noted<br>*Give P values as exact values whenever suitable.*
☒	☐	For Bayesian analysis, information on the choice of priors and Markov chain Monte Carlo settings
☒	☐	For hierarchical and complex designs, identification of the appropriate level for tests and full reporting of outcomes
☒	☐	Estimates of effect sizes (e.g. Cohen's d, Pearson's r), indicating how they were calculated

Our web collection on statistics for biologists contains articles on many of the points above.

Software and code

Policy information about availability of computer code

Data collection	Whole and targeted exome sequencing: Nimblegen V2 or Agilent V5 platform and Illumina HiSeq 2000 sequencing
Data analysis	Whole and targeted exome sequencing: BWA-MEM [bwa-0.7.12], picard-tools-1.119, GATK-3.4-46<br>Open sources: Picard Tools v1.141 (CollectWgsMetrics), gnomAD database v2.0.2, R version 3.4.4.<br>All tools and scripts used for processing the WES data can be provided by authors upon request.

For manuscripts utilizing custom algorithms or software that are central to the research but not yet described in published literature, software must be made available to editors and reviewers. We strongly encourage code deposition in a community repository (e.g. GitHub). See the Nature Portfolio guidelines for submitting code & software for further information.

Data

Policy information about availability of data

All manuscripts must include a data availability statement. This statement should provide the following information, where applicable:

- Accession codes, unique identifiers, or web links for publicly available datasets
- A description of any restrictions on data availability
- For clinical datasets or third party data, please ensure that the statement adheres to our policy

The whole exome sequencing datasets generated during and/or analysed during the current study are available at DOI: 10.6084/m9.figshare.14778894

Field-specific reporting

Please select the one below that is the best fit for your research. If you are not sure, read the appropriate sections before making your selection.

☒ Life sciences ☐ Behavioural & social sciences ☐ Ecological, evolutionary & environmental sciences

For a reference copy of the document with all sections, see nature.com/documents/nr-reporting-summary-flat.pdf

Life sciences study design

All studies must disclose on these points even when the disclosure is negative.

Sample size	Data from all available familial delayed puberty pedigrees available were recruited, without performing sample-size calculation. To do any power calculation, one needs to have some established data from literature to estimate effects. We deemed 100 proband samples sufficient for the analyses described.
Data exclusions	There were no exclusions for the whole or exome sequencing study.
Replication	The whole exome sequencing study has not been replicated as there are no other large cohorts of patients with the same phenotype currently available.
Randomization	N/A
Blinding	N/A

Reporting for specific materials, systems and methods

We require information from authors about some types of materials, experimental systems and methods used in many studies. Here, indicate whether each material, system or method listed is relevant to your study. If you are not sure if a list item applies to your research, read the appropriate section before selecting a response.

Materials & experimental systems

n/a	Involved in the study	
☐	☒	Antibodies
☐	☒	Eukaryotic cell lines
☒	☐	Palaeontology and archaeology
☒	☐	Animals and other organisms
☐	☒	Human research participants
☒	☐	Clinical data
☒	☐	Dual use research of concern

Methods

n/a	Involved in the study	
☒	☐	ChIP-seq
☒	☐	Flow cytometry
☒	☐	MRI-based neuroimaging

Antibodies

Antibodies used	CCDC141 (SAB3500670, Sigma Aldrich), GAPDH (G9545, Sigma Aldrich and MA5-15738, Thermofisher), FLAG (F1804, Sigma Aldrich), Ac-tubulin (ab179484, Abcam), AC-pericentrin (ab4448, Abcam)
Validation	Antibody validation for Western blotting: negative controls were loaded (i.e. not transfected HEK293T cells).<br>Antibody validation for Immunohistochemistry/Immunofluorescence: one slide was incubated with blocking buffer only (no primary antibody) and then incubated with secondary antibodies.

Eukaryotic cell lines

Policy information about cell lines

Cell line source(s)	HEK293T: ATCC® CRL-3216™
Authentication	The cell line was not authenticated in our laboratory
Mycoplasma contamination	The cell line tested negative for Mycoplasma contamination. Cells were tested every months. (MycoAlert Detection Kit, Lonza).
Commonly misidentified lines (See ICLAC register)	N/A

Human research participants

Policy information about studies involving human research participants

Population characteristics	All patients were referred to specialist paediatric care in central and southern Finland (1982-2004). All patients met the diagnostic criteria for self-limited delayed puberty, defined as the onset of Tanner genital stage II (testicular volume >3 ml) >13.5yr in boys or Tanner breast stage II >13.0yr in girls (i.e. two SD later than average pubertal development). Chronic illness and undernutrition was excluded by medical history, clinical examination, and routine laboratory tests. GnRH deficiency, if suspected, was excluded by spontaneous pubertal development at follow-up.
Recruitment	Patients referred with delayed puberty to specialist paediatric care in central and southern Finland (1982-2004) were identified and offered recruitment. Families of the DP patients were invited to participate, with information about medical history and pubertal timing obtained by structured interviews and from archived height records.
Ethics oversight	The study protocol was approved by the Ethics Committee for Pediatrics, Adolescent Medicine and Psychiatry, Hospital District of Helsinki and Uusimaa (570/E7/2003). UK ethical approval was granted by the London-Chelsea NRES committee (13/LO/0257). The study was conducted in accordance with the guidelines of The Declaration of Helsinki.

Note that full information on the approval of the study protocol must also be provided in the manuscript.
