# Supplementary material for: Whole exome sequencing identifies deleterious rare variants in CCDC141 in familial self-limited delayed puberty
Source: NPJ Genom Med. 2021 Dec 20;6:107. doi: 10.1038/s41525-021-00274-w (PMC8688425; doi:10.1038/s41525-021-00274-w)
Supplement: Supplementary file 2 — Supplementary Information [file 41525_2021_274_MOESM2_ESM.pdf]

Supplementary Data

Supplementary Figures

A

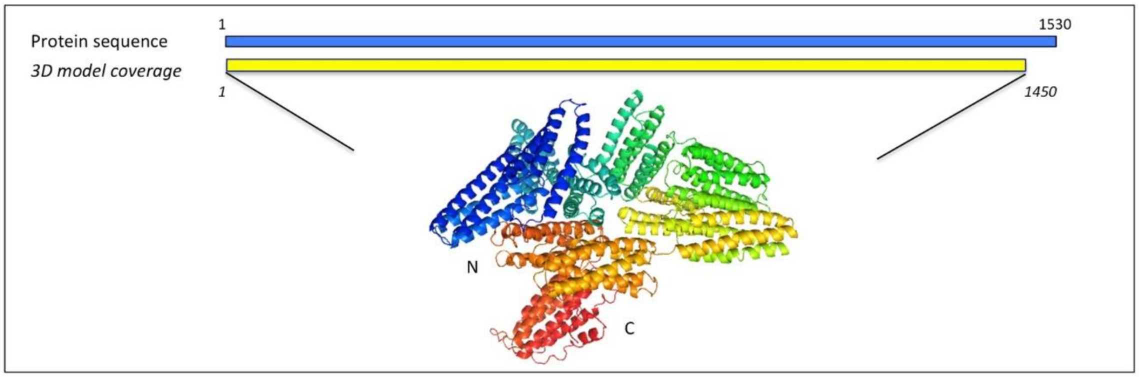

B

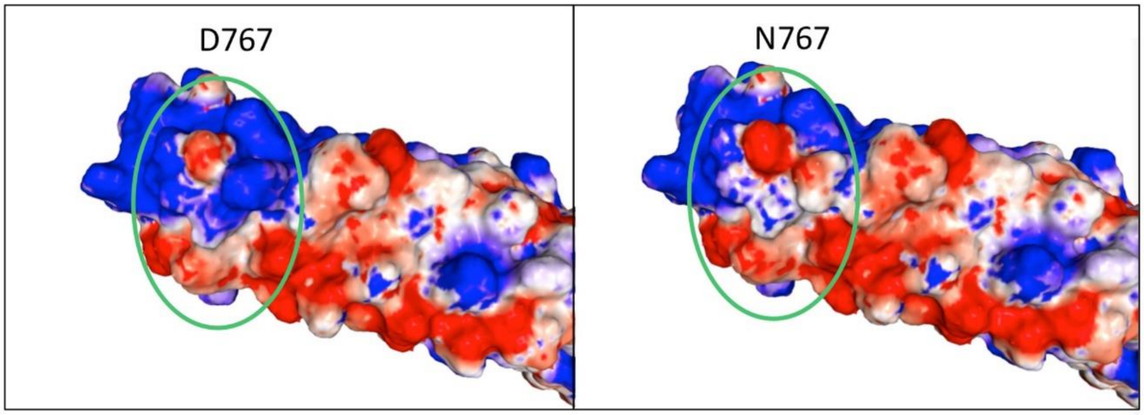

Supplementary Figure 1 Schematic representation of the CCDC141 3D model. **A)** Globular conformation of CCDC141. Cartoon representation of CCDC141 3D model structure generated by the I-Tasser homology modelling software. Rainbow colouring from blue to red is used to show the protein topology from the N- to C-terminus. The blue and yellow bars above the 3D structure represent the CCDC141 amino acid sequence and amino acid region covered by the model. **B)** Example of p.D767N variant which shows the solvent-accessible surface with electrostatic potential. The wild type CCDC141 structures are presented on the left and the

mutant structures on the right. The isoelectric potential +1 kcal/(mol·e) is presented in blue and −1 kcal/(mol·e) in red.

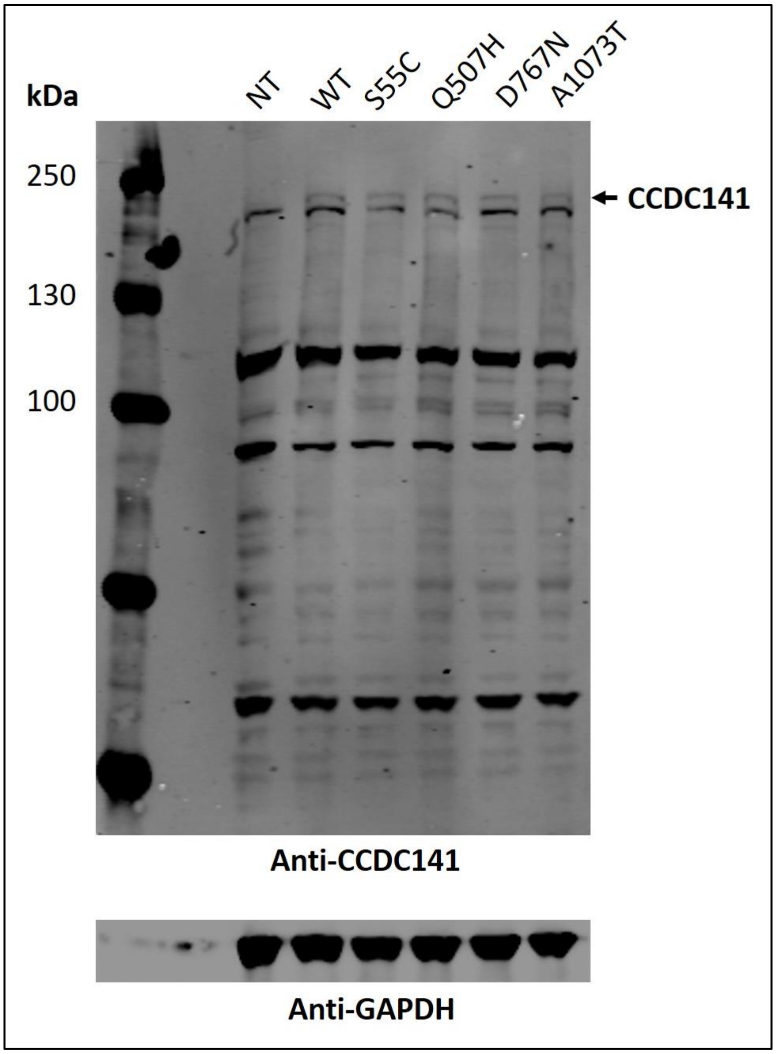

Supplementary Figure 2. Western blot analysis of expressed WT and CCDC141 mutants in HEK293T cells, blotted with anti-CCDC141 (top panel) and anti-GAPDH (bottom panel) antibodies. NT are not transfected HEK293T cells as a control. CCDC141 is visible as a single band of approximately 170 kDa (expected size: 166 kDa).

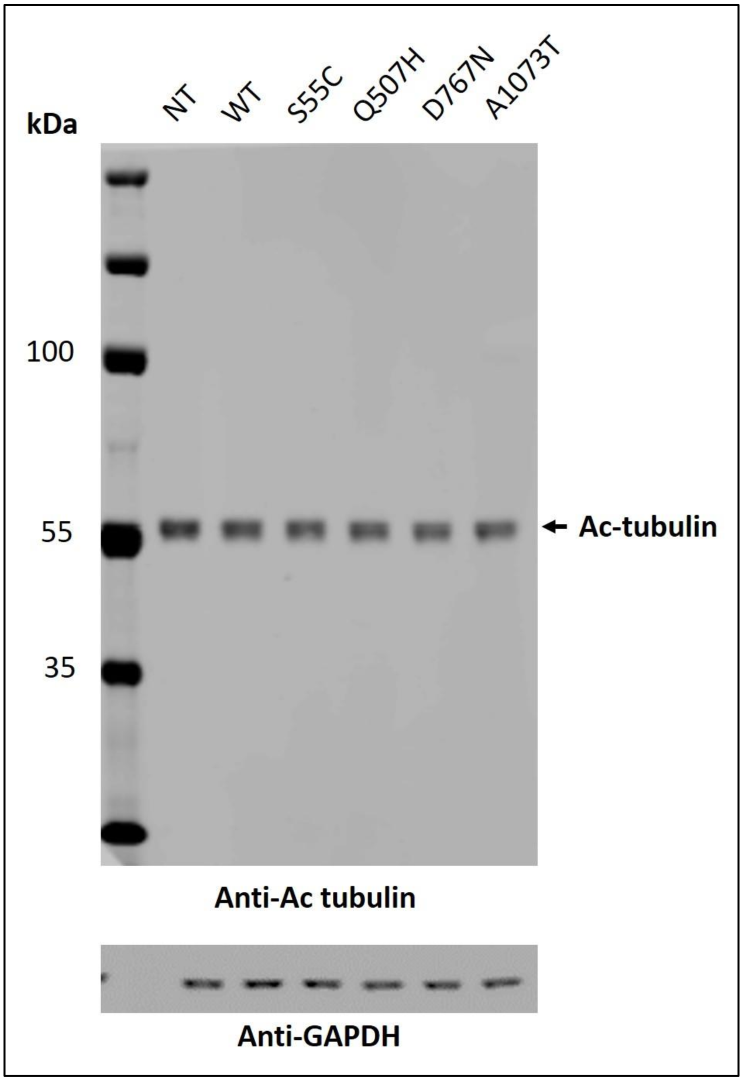

Supplementary Figure 3 Expression of CCDC141 mutants does not alter total Ac-tubulin levels in cell lysates. Level of Ac-tubulin and GAPDH in HEK293 cells stably expressing CCDC141 were demonstrated by Western blotting. NT – non-transfected HEK293 cells as a control.

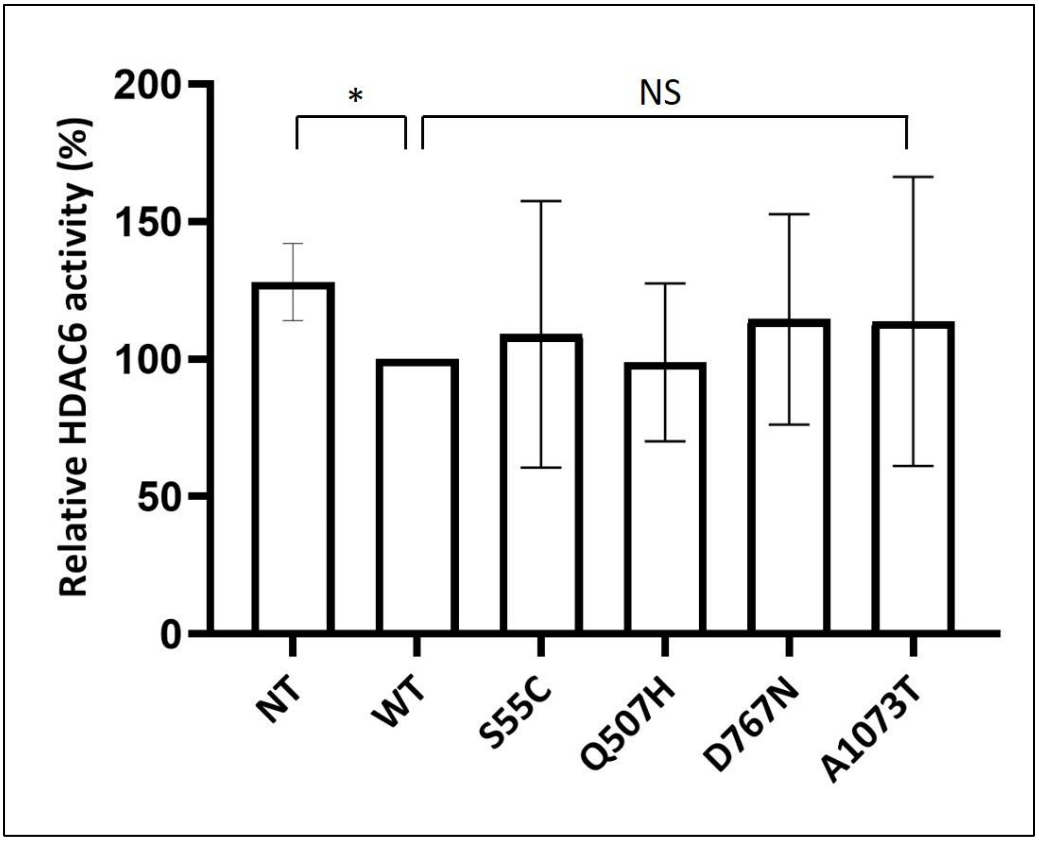

Supplementary Figure 4 Overexpression of CCDC141 decreases HDAC6 activity in transiently transfected HEK293T cells, but HDAC6 activity in HEK293T cells expressing mutant CCDC141 proteins is not significantly different from HEK293T cells expressing the WT CCDC141. * $p<0.05$; NS-not significant; NT-non-transfected HEK293T cells.

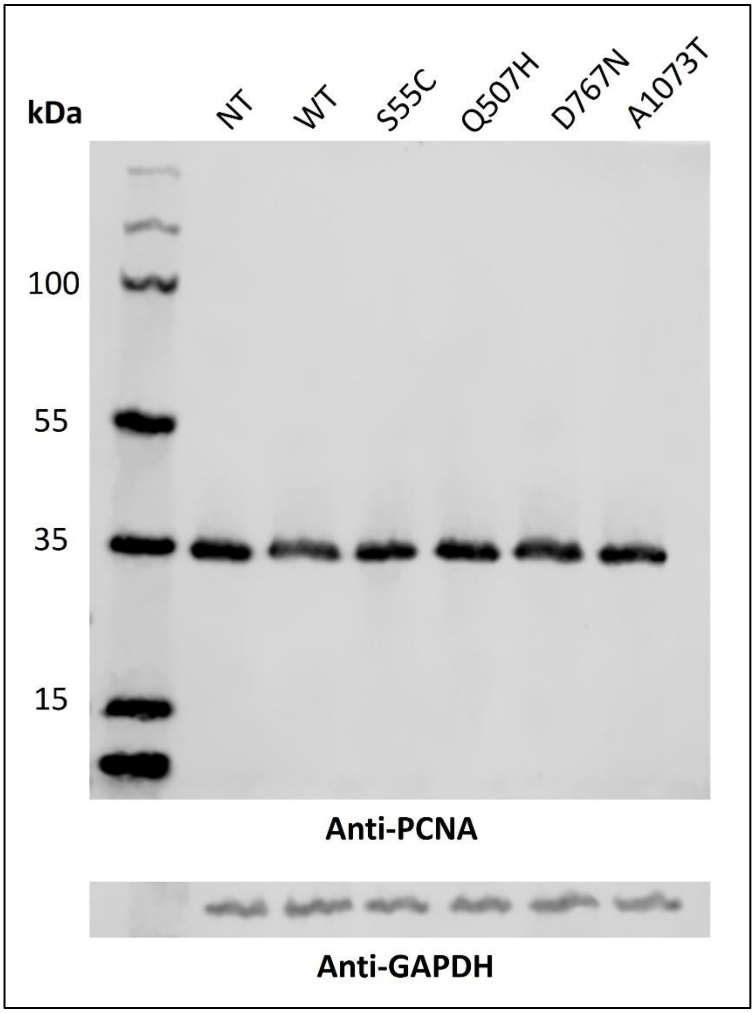

Supplementary Figure 5. Proliferation of HEK293T cells expressing CCDC141 proteins is not different between WT and mutants. NT-non-transfected HEK293T cells as a control. Level of PCNA and GAPDH in HEK293 cells stably expressing CCDC141 as demonstrated by Western blotting.

Gene	DP_VAR	DP_allele	gnomAD_Finn_VAR	gnomAD_Finn_allele	gnomAD_p.value	gnomAD_FDR
CCDC141	6	200	332	25120	0.052	0.209581
POLR3B	5	200	245	25168	0.048	0.209581
DCC	3	200	193	25120	0.202	0.404848
PLXNA1	2	200	516	25120	0.448	0.597406
FEZF1	1	200	134	25104	1	1
SEMA3E	1	200	160	25120	1	1
KLB	1	200	572	25120	0.143	0.380659
NTN1	1	200	447	25120	0.274	0.437608
PNPLA6	0	200	-	-	-	-
STUB1	0	200	-	-	-	-
DMXL2	0	200	-	-	-	-
SMCHD1	0	200	-	-	-	-

Supplementary Table 1. Results of Whole Gene Rare Variant Burden Testing for all rare (total population minor allele frequency (MAF) <1%) and predicted deleterious variants in the 12 candidate genes in the gnomAD database (accessed Aug 2021). Rare, predicted deleterious variants in *CCDC141* were found more commonly in self-limited delayed puberty subjects as compared to the gnomAD Finnish control populations. In 4 of the 12 candidate genes (PNPLA6, STUB1, DMXL2, SMCHD1) no rare and predicted deleterious variants were seen in the DP cohort. DP_VAR – number of rare, predicted deleterious variants found in that gene in the DP cohort; DP_Allele – number of alleles sequenced in the DP cohort (for 100 probands); gnomAD_Finn_VAR - number of rare, predicted deleterious variants found in that gene in the Finnish gnomAD dataset; gnomAD_Finn_Allele - number of alleles sequenced for that gene in the Finnish gnomAD dataset; gnomAD_FDR – gnomAD p value after FDR multiple comparison adjustment.

Variants	Forward primers	Reverse primers
p.Ser55Cys	TTCTAGAAATTGGCTGCAG TCAAGATGAA	TTCATCTTGACTGCAGCCAAT TTCTAGAA
p.Gln507His	GAACTAGATATCCATGCTA AGGAGACATC	GATGTCTCCTTAGCATGGATA TCTAGTTC
p.Asp767Asn	CTCAACAACTGAAGAACCT TATTCACTTC	GAAGTGAATAAGGTTCTTCAG TTGTTGAG
p.Asn926Ile	TAATTTGAAGTTTATTTAC ACTAAGAAAA	TTTTCTTAGTGTAAATAAACT TCAAATTA
p.Ala1073Thr	AAAGGATTCAGGAGACCA CTGACCTTGCT	AGCAAGGTCAGTGGTCTCCTG AATCCTTT

Supplementary Table 2. Primers used for site directed mutagenesis of *CCDC141* variants

Variants	Forward primers	Reverse primers
p.Ser55Cys	GTTGCGCTTTCTACGACGAC	GGGAACTCTTGCAGGAAGCA
p.Gln507His	AGTGGAGGGTTACCTACGGA	AGATCGCAAGTCCTGCAAAC
p.Asp767Asn	CCTGTGTCTGCACTTGACCT	TGGAGTTTGTAGAGCAGCCG
p.Asn926Ile, p.Ala1073Thr	CGAAGATGCAAGTGCCACAG	TCCCTCACAGAGCTCGAAGA

Supplementary Table 3. Primers used to sequence *CCDC141* variants in vector cDNA
